# Performance Analysis of Amplify-and-Forward Systems with Single Relay Selection in Correlated Environments

**DOI:** 10.3390/s16091472

**Published:** 2016-09-11

**Authors:** Binh Van Nguyen, Kiseon Kim

**Affiliations:** 1 Department of Nanobio Materials and Electronics, Gwangju Institute of Science and Technology, Gwangju 61005, Korea; binhnguyen@gist.ac.kr; 2 School of Electrical Engineering and Computer Science, Gwangju Institute of Science and Technology, Gwangju 61005, Korea

**Keywords:** cooperative sensor systems, amplify-and-forward, correlated environments, outage probability, diversity order

## Abstract

In this paper, we consider amplify-and-forward (AnF) cooperative systems under correlated fading environments. We first present a brief overview of existing works on the effect of channel correlations on the system performance. We then focus on our main contribution which is analyzing the outage probability of a multi-AnF-relay system with the best relay selection (BRS) scheme under a condition that two channels of each relay, source-relay and relay-destination channels, are correlated. Using lower and upper bounds on the end-to-end received signal-to-noise ratio (SNR) at the destination, we derive corresponding upper and lower bounds on the system outage probability. We prove that the system can achieve a diversity order (DO) equal to the number of relays. In addition, and importantly, we show that the considered correlation form has a constructive effect on the system performance. In other words, the larger the correlation coefficient, the better system performance. Our analytic results are corroborated by extensive Monte-Carlo simulations.

## 1. Introduction

Cooperative communication is of considerable interest due to its ability to provide the benefits of spatial-diversity to single antenna systems. It is based on the shared nature of the wireless medium in which a number of distributed neighbors, called relays, can overhear a source’s transmission and forward a manipulated version of their received signal to an intended destination. Among various relaying protocols, the two most popular are amplify-and-forward (AnF) and decode-and-forward (DnF) [1,2]. In AnF systems, single or multiple relays simply amplify and then forward their received signals to the destination. On the other hand, in DnF systems, single or multiple relays decode, re-encode, and forward the outcome to the destination. In multi-relay cooperative systems, single relay selection (SRS) is of importance for practical implementation because it not only reduces the system complexity but also improves the system spectral efficiency while maintaining the system diversity order (DO) compared to schemes exploiting all available relays, i.e., orthogonal transmission, distributed space-time coding, and distributed beamforming schemes [2]. SRS investigated in existing literature can be categorized as best relay selection (BRS) and partial relay selection (PRS) [3,4,5,6]. In BRS, a single relay is selected based on the instantaneous global (two hops) channel state information (CSI), while in PRS, only local (single hop) CSI is used to activate a relay. It is obvious that the BRS provides a better system performance than that of the PRS counterpart. However, the PRS scheme incurs much lower deployment complexity, and thus, is highly preferable for systems with complexity constraints.

**Figure 1:**
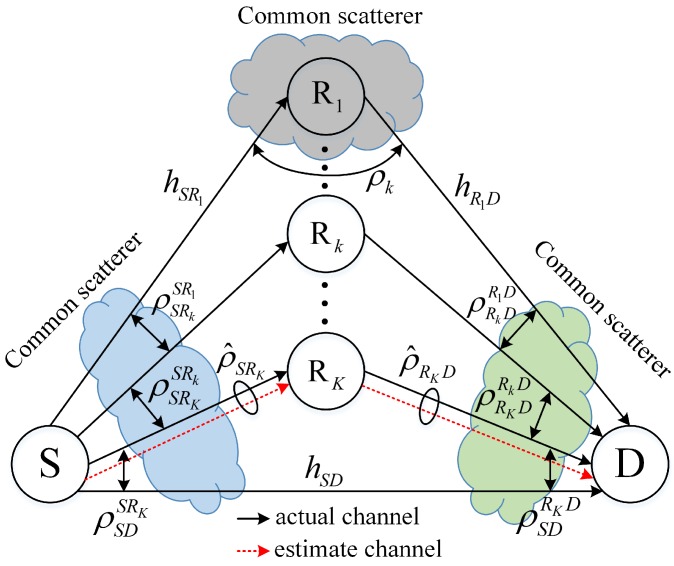
A multi-relay system in a general correlated environment.

Although SRS and related performance analysis have been extensively investigated, most of the existing works assume that wireless channels are statistically independent. However, in reality, wireless channels are likely to be correlated due to feedback delay, common scatterers, geometry of the channels, or characteristics of the channels [7,8,9]. Particularly, in wireless cooperative sensor networks, nodes are commonly low-complexity and densely deployed. Therefore, feedback delay and common scatterers among nodes highly exist. An illustration of a general cooperative system, in which a source *S* communicates with a destination *D* via *K* number of relays, ${\left\{R_{k}\right\}}_{k=1}^{K}$, in a correlated environment, is given in Figure 1. In the given system, correlations may exist between the source-relay (first-hop) channels, between the relay-destination (second-hop) channels, between the actual and the corresponding estimate channels, between the source-$R_{k}$ and $R_{k}$-destination channels, or between the source-destination and source-relay (relay-destination) channels. A detailed description of channel correlations is given in Table 1.

Here in this work, we first review existing works on the effects of channel correlations on the performance of AnF systems. We then focus on our main contribution, which is to analyze the effect of the channel correlation between the two channels of each relay (source-$R_{k}$ and $R_{k}$-destination channels) on the performance of a multiple AnF relay system with the BRS scheme. We quantify the system DO which is the foremost important performance measure that characterises the system behaviour in the signal-to-noise ratio (SNR) region. In addition, and importantly, from the system DO, we reveal novel insights on the impact of the considered correlation on the system performance. Particularly, we prove that the considered correlation does not affect the system DO. Moreover, we show that the considered correlation has a constructive effect on the system performance.

The remainder of this paper is organized as follows. Section 2 introduces a general system model. A brief overview of existing works on channel correlations is given in Section 3. In Section 4, we present our investigation for the effect of the channel correlation between two channels of each relay on the system performance. Simulation results are provided in Section 5, followed by our conclusion and discussion on future research directions in Section 6.

## 2. General System Model

A general cooperative system in correlated environment is graphically depicted in Figure 1. The system consists of a source *S*, a destination *D*, and *K* number of relays, which are denoted as ${\left\{R_{k}\right\}}_{k=1}^{K}$. All the nodes have a single antenna that can be used for both transmission and reception. All the relays are in the half-duplex mode, and thus, cannot transmit and receive simultaneously; they also employ the variable-gain AnF relaying protocol.

The channel gain between node *i* and *j* is denoted as $h_{ij}$. The noise associated with each channel is modeled as a mutually independent additive white Gaussian noise (AWGN) with zero-mean and variance $N_{0}$. We do not consider power allocation issues as they are outside the scope of this work. We assume that each transmitter (source or relay) transmits information with a fixed power *P*. We use the notation $\gamma_{ij}=\frac{P}{N_{0}}{\left|h_{ij}\right|}^{2}$ to refer to the instantaneous SNR of link i−j. Additionally, we use $f_{X}\left(\cdot \right)$ and $F_{X}\left(\cdot \right)$ to denote the probability density function (PDF) and the cumulative density function (CDF) of a random variable *X*, respectively. Furthermore, the notation $\mathrm{Pr}\left[Y\right]$ is the probability of an event *Y*.

Depending on the characteristic or geometry of the channels, channel correlations may exist. For instance, due to feedback or scheduling delays, the channel state of the selected relay at the selection decision instant can substantially differ from the actual channel during its data forwarding [10,11]. As a result, the correlation, ${\hat{\rho }}_{ij}$, exists between the actual channel ($h_{ij}$) and the corresponding estimate (${\hat{h}}_{ij}$). In other words, $h_{ij}$ and ${\hat{h}}_{ij}$ are correlated random variables (RVs) with the following correlation matrix:

(1) $$\begin{array}{c} CR_{1}=\left(\begin{array}{cc} 1 & {\hat{\rho }}_{ij}\\ {\hat{\rho }}_{ij} & 1 \end{array}\right). \end{array}$$

Alternatively, if there exist common scatterers near the source, the first-hop and the source-destination channels are likely to be correlated to each other. The reason is that nearby channels are often affected by the same scatterers (shadowers); therefore, channels’ losses are correlated [12,13,14]. Consequently, ${\left\{h_{SR_{k}}\right\}}_{k=1}^{K}$ and $h_{SD}$ may be modelled as correlated RVs with the following correlation matrix:

(2) $$\begin{array}{c} CR_{2}=\left(\begin{array}{ccccc} 1 & \rho_{SR_{2}}^{SR_{1}} & \ldots & \rho_{SR_{K}}^{SR_{1}} & \rho_{SD}^{SR_{1}}\\ \rho_{SR_{2}}^{SR_{1}} & 1 & \ldots & \rho_{SR_{K}}^{SR_{2}} & \rho_{SD}^{SR_{2}}\\ \vdots & \vdots & \ddots & \vdots & \vdots \\ \rho_{SR_{K}}^{SR_{1}} & \rho_{SR_{K}}^{SR_{2}} & \ldots & 1 & \rho_{SD}^{SR_{K}}\\ \rho_{SD}^{SR_{1}} & \rho_{SD}^{SR_{2}} & \ldots & \rho_{SD}^{SR_{K}} & 1 \end{array}\right). \end{array}$$

Similarly, ${\left\{h_{R_{k}D}\right\}}_{k=1}^{K}$ and $h_{SD}$ may also be correlated RVs with a correlation matrix given by

(3) $$\begin{array}{c} CR_{3}=\left(\begin{array}{ccccc} 1 & \rho_{R_{2}D}^{R_{1}D} & \ldots & \rho_{R_{K}D}^{R_{1}D} & \rho_{SD}^{R_{1}D}\\ \rho_{R_{2}D}^{R_{1}D} & 1 & \ldots & \rho_{R_{K}D}^{R_{2}D} & \rho_{SD}^{R_{2}D}\\ \vdots & \vdots & \ddots & \vdots & \vdots \\ \rho_{R_{K}D}^{R_{1}D} & \rho_{R_{K}D}^{R_{2}D} & \ldots & 1 & \rho_{SD}^{R_{K}D}\\ \rho_{SD}^{R_{1}D} & \rho_{SD}^{R_{2}D} & \ldots & \rho_{SD}^{R_{K}D} & 1 \end{array}\right), \end{array}$$

when there are common scatterers in the vicinity of the destination. Moreover, in indoor systems or in systems in which the source-$R_{k}$ and the $R_{k}$-destination channels are impinged by the same scatterers, the two channels of the relay $R_{k}$ can also be correlated with the following correlation matrix [8,9,13,15]:

(4) $$\begin{array}{c} CR_{4}=\left(\begin{array}{cc} 1 & \rho_{k}\\ \rho_{k} & 1 \end{array}\right). \end{array}$$

Because the relays operate in the half-duplex mode, each source-destination communication is divided into two consecutive phases. In the first phase, the source broadcasts its signal to the relays and the destination (if the direct link is available). In the second phase, the selected relay forwards an amplified version of its received signal to the destination. Suppose that *S* communicates with *D* through the relay $R_{k}$, then the total end-to-end SNR at *D* is given by [1]

(5) $$\begin{array}{c} \gamma_{D}^{T}=\gamma_{SD}+\gamma_{SR_{k}D}, \end{array}$$

where $\gamma_{SD}=\frac{P}{N_{0}}{\left|h_{SD}\right|}^{2}$ and

(6) $$\begin{array}{cc} \frac{1}{2}\min\left\{\gamma_{SR_{k}},\gamma_{R_{k}D}\right\}\leq \gamma_{SR_{k}D}=\frac{\gamma_{SR_{k}}\gamma_{R_{k}D}}{\gamma_{SR_{k}}+\gamma_{R_{k}D}+1} & \leq \min\left\{\gamma_{SR_{k}},\gamma_{R_{k}D}\right\}. \end{array}$$

In the BRS scheme, the relay that maximizes the bottleneck link is selected. Mathematically, the scheme is expressed as [4]

(7) $$\begin{array}{c} b=\underset{k=1,\cdots ,K}{\arg\;\max}\left\{\min\left\{\gamma_{SR_{k}},\gamma_{R_{k}D}\right\}\right\}. \end{array}$$

On the other hand, in the PRS scheme, the relay with the strongest SNR over the source-relay (or relay-destination) link is activated, which is [3]

(8) $$\begin{array}{c} p=\underset{k=1,\ldots ,K}{\arg\;\max}\left[\gamma_{SR_{k}}\right], \end{array}$$

or

(9) $$\begin{array}{c} p=\underset{k=1,\cdots ,K}{\arg\;\max}\left[\gamma_{R_{k}D}\right]. \end{array}$$

In Equations (7)–(9), *b* and *p* are the indexes of the selected relays. It is also important to note that, sometimes, the best relay (according to either the BRS or the PRS schemes) may not be available to assist the source-destination transmission due to some scheduling schemes, loads balancing conditions, or insufficient transmit power [16]. Consequently, we have to select the $l^{th}$ best relay instead. This relay selection criterion is referred as the $l^{th}$-BRS and $l^{th}$-PRS in the sequel.

The previously presented relay selection schemes can be deployed following a centralized or a distributed approach. In a centralized approach, a central node (source, destination, or another dedicated node) maintains a table that contains all the relays with associated channel gains. Using the table, the best relay can be easily selected. In a distributed approach, each relay keeps a timer, whose initial value is inversely proportional to $\min\left\{\gamma_{SR_{k}},\gamma_{R_{k}D}\right\}$ for the BRS scheme, and $\gamma_{SR_{k}}$ (or $\gamma_{R_{k}D}$) for the PRS scheme. Then, the selected relay will be the one that has the smallest initial value of the timer [6].

## 3. An Overview of Existing Works

Before focusing on our main analysis, we first go over the existing works that tackle the effect of channel correlations on the performance of AnF cooperative systems. A summary of channel correlation models considered in the existing literature is given in Table 2.

### 3.1. Correlation between the Actual and the Corresponding Estimate Channels

**Figure 2:**
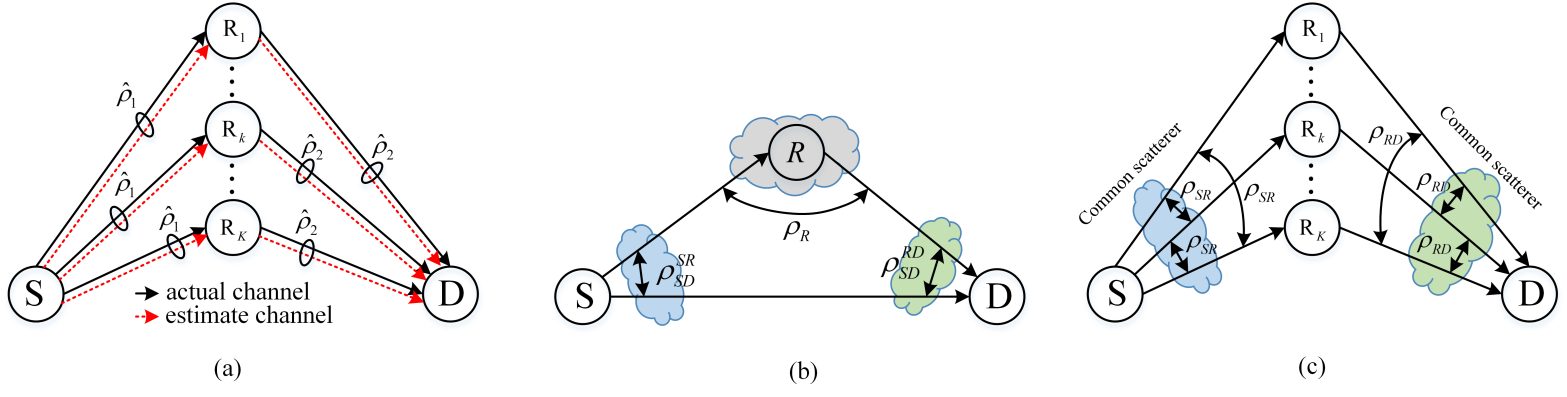
Cooperative systems in correlated environments have been investigated in (**a**) [17,18,19]; (**b**) [20]; and (**c**) [21].

The considered system model is referred to as Model A in Table 2 and illustrated in Figure 2a, in which the direct link between the source and the destination is assumed to be unavailable. The end-to-end SNR at the destination with the support of the relay $R_{k}$ is given in Equation (6). In [17,18,19], the PRS and the BRS, and the $l^{th}$-PRS and the $l^{th}$-BRS schemes are considered, respectively. More specifically, a single relay is selected based on outdated channels, which is

(10) $$\begin{array}{c} b=\underset{k=1,\cdots ,K}{\arg\;\max}\{\min\{{\hat{\gamma }}_{SR_{k}},{\hat{\gamma }}_{R_{k}D}\}\}, \end{array}$$

for the BRS ($l^{th}$-BRS) scheme. In addition,

(11) $$\begin{array}{c} p=\underset{k=1,\ldots ,K}{\arg\;\max}[{\hat{\gamma }}_{SR_{k}}] \end{array}$$

for PRS ($l^{th}$-PRS) scheme, where ${\hat{\gamma }}_{ij}=\frac{P}{N_{0}}{\left|{\hat{h}}_{ij}\right|}^{2}$ denotes the (outdated) estimate of $\gamma_{ij}$. We should note that the estimate end-to-end SNR at the destination with the support of the selected relay is different from what the destination is supposed to receive. The authors then derive the system performances, i.e., outage probability and symbol error probability, under Rayleigh fading and show that, when CSI is outdated, the system DO is reduced to one. They also show that a 3% reduction in correlation causes up to an order of magnitude increase in the system outage probability. They then suggest that a high feedback rate may be required in practice in order to attain the full benefits of the BRS scheme. Moreover, it is shown that, depending on the correlation value, the PRS scheme may outperform the BRS scheme and vice versa. However, it is worth pointing out that these works are limited to the case of equally correlation coefficients, i.e., ${\left\{{\hat{\rho }}_{SR_{k}}\right\}}_{k=1}^{K}={\hat{\rho }}_{1}$ and ${\left\{{\hat{\rho }}_{R_{k}D}\right\}}_{k=1}^{K}={\hat{\rho }}_{2}$. The systems with different correlation coefficients have not been investigated yet.

### 3.2. Correlations between Source-Relay and Relay-Destination Channels, between Source-Destination and Source-Relay Channels, and between Source-Destination and Relay-Destination Channels

The investigated system is illustrated in Figure 2b and Model B in Table 2, where only a single relay case is considered. The direct source-destination link is also taken into account. The total end-to-end SNR at the destination is given in Equation (5) by dropping the index *k*. The authors of [20] consider the impact of each channel correlation on the system performance separately. The moment generating function of $\gamma_{D}^{T}$, $M_{\gamma_{D}^{T}}\left(s\right)$ is first derived in closed-form under conditions of Nakagami-*m* fading. Consequently, the bit error rate of the differential binary phase shift keying is obtained as $M_{\gamma_{D}^{T}}\left(1\right)$. They show that all three of the correlation forms degrade the system performance. Moreover, they also point out that the effect of correlation is often noticeable when the correlation coefficient is larger than 0.5. However, this work is limited for single relay systems only. Additionally, considering all the three correlation forms simultaneously also needs further investigation.

### 3.3. Correlation between First-Hop Channels and Correlation between Second-Hop Channels

Recently, Nguyen et al. has studied the joint impact of the correlations between the first-hop channels and between the second-hop channels on the performance of a multiple AnF relay cooperative system with the BRS scheme [21]. The system model is schematically illustrated in Figure 2c, in which the source-destination channel is assumed to be unavailable. The assumption on the channel correlations is shown in Table 2, Model C. More specifically, the authors assume that ${\left\{\rho_{SR_{k}}^{SR_{l}}\right\}}_{\begin{array}{l} l,k=1\\ l\neq k \end{array}}^{K}=\rho_{SR}$ and ${\left\{\rho_{R_{k}D}^{R_{l}D}\right\}}_{\begin{array}{l} l,k=1\\ l\neq k \end{array}}^{K}=\rho_{RD}$. By exploiting the axioms of probability and the principle of inclusion and exclusion for probability, the authors derive a lower bound on the system outage probability under Rayleigh fading. In order to gain further insights on the effect of the considered correlation on the system DO, the authors provide a closed-form expression as well as the corresponding asymptotic approximation of the outage probability of a two-relay system. They point out that even though the system performance is considerably degraded in the high SNR region when the correlation coefficients are sufficiently large, yet less than one, the system still achieves a DO equal to the number of relays. We should keep in mind that [21] considers the identical correlation coefficient case, and the cases with unequal channel correlation coefficients still remain unsolved.

## 4. An Investigation for a Remaining Issue

Even though there exist several interesting open problems related to channel correlations that have not been reported in the literature, it is of our interest to analyze the effect of the correlation between the two channels of each relay on the performance of a multiple AnF relays system with the BRS scheme. In other words, we extend the work presented in [20] to tackle a multi-relay scenario with the BRS scheme. The problem is motivated by the fact that, in reality, multiple relays can be available. In addition, in multi-relay systems, a SRS scheme is commonly utilized to enhance the systems performance with affordable deployment complexity.

### 4.1. System Model

**Figure 3:**
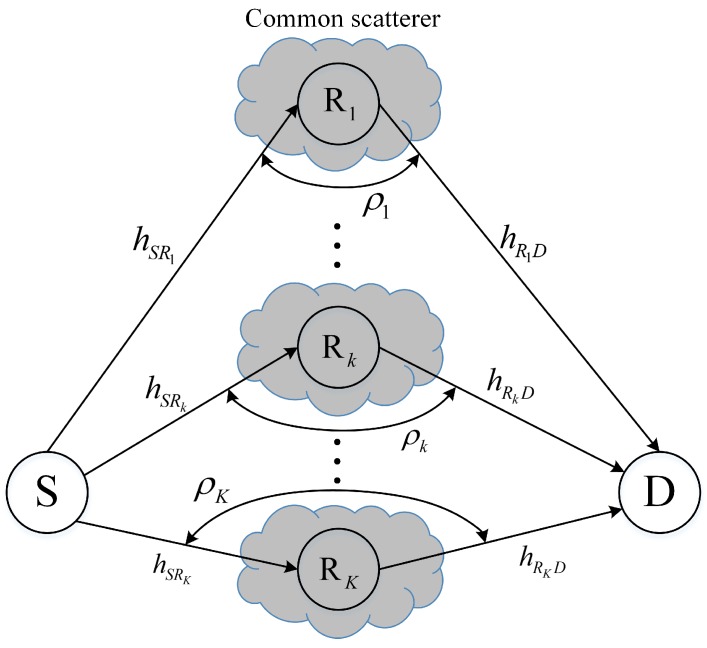
A multiple half-duplex AnF system in the considered correlated environment.

The system model is given in Figure 3, in which the direct link is assumed to be unavailable, which may be a result of severe fading and shadowing between the source and destination. In this network, we assume that all channels are subject to block Rayleigh fading, which is when the fading keeps constant during a transmission block and changes independently from one to another. For each relay $R_{k}$, its channels, $h_{SR_{k}}$ and $h_{R_{k}D}$, are modelled as correlated zero-mean complex Gaussian RVs with variance $\delta_{k}^{2}$. In addition, for any pair of relays $R_{l}$ and $R_{k}$, their channels are independent. It is noteworthy again that the assumption of the correlation between the two channels of each relay is justifiable when (1) there are common scatterers that affect both $h_{SR_{k}}$ and $h_{R_{k}D}$ [9,14,15]; (2) the relays are moving between the source and the destination [9,22]; and (3) when the considered system is deployed in an indoor environment [8]. The end-to-end SNR at the destination of the selected relay is given by

(12) $$\begin{array}{c} \gamma_{SR_{b}D}=\frac{\gamma_{SR_{b}}\gamma_{R_{b}D}}{\gamma_{SR_{b}}+\gamma_{R_{b}D}+1}, \end{array}$$

which is bounded as follows [23]

(13) $$\begin{array}{c} \max_{k=1,\cdots ,K}\left[\frac{1}{2}\min\left(\gamma_{SR_{k}},\gamma_{R_{k}D}\right)\right]\leq \gamma_{SR_{b}D}\leq \max_{k=1,\cdots ,K}\left[\min\left(\gamma_{SR_{k}},\gamma_{R_{k}D}\right)\right]. \end{array}$$

### 4.2. Outage Probability Analysis

The system outage probability is defined as the probability of the event that the instantaneous system capacity, $C=\frac{1}{2}\log_{2}\left(1+\gamma_{SR_{b}D}\right)$, is less than a predefined threshold $R_{0}$ in bits/s/Hz

(14) $$\begin{array}{cc} P_{out} & =\mathrm{Pr}\left[C<R_{0}\right]\\ & =\mathrm{Pr}\left[\gamma_{SR_{b}D}<2^{2R_{0}}-1\right]\\ & =F_{\gamma_{SR_{b}D}}\left(2^{2R_{0}}-1\right), \end{array}$$

where $R_{0}$ is the system spectral efficiency in bits/s/Hz. As deriving an exact expression for the system outage probability is cumbersome, for a more tractable analysis, we start with the bounds on the end-to-end received SNR given in Equation (13). The CDF of $\min\left(\gamma_{SR_{k}},\gamma_{R_{k}D}\right)$, $F_{\min}^{k}\left(\gamma \right)$, is formulated as follows

(15) $$\begin{array}{cc} F_{\min}^{k}\left(\gamma \right) & =\mathrm{Pr}\left[\min\left(\gamma_{SR_{k}},\gamma_{R_{k}D}\right)\leq \gamma \right]\\ & =2-\mathrm{Pr}\left[\gamma_{SR_{k}}>\gamma \right]-\mathrm{Pr}\left[\gamma_{R_{k}D}>\gamma \right]-\mathrm{Pr}\left[\gamma_{SR_{k}}\leq \gamma ,\gamma_{R_{k}D}\leq \gamma \right], \end{array}$$

where the marginal probabilities $\mathrm{Pr}\left[\gamma_{SR_{k}}>\gamma \right]$ and $\mathrm{Pr}\left[\gamma_{R_{k}D}>\gamma \right]$ are equal to $e^{-\frac{\gamma }{\lambda_{k}}}$. Making use of Equation (16) in [13], we obtain

(16) $$\begin{array}{cc} \mathrm{Pr}\left[\gamma_{SR_{k}}\leq \gamma ,\gamma_{R_{k}D}\leq \gamma \right]= & 1+e^{\frac{-2\gamma }{\lambda_{k}\left(1-\rho_{k}^{2}\right)}}I_{0}\left(\frac{2\rho_{k}\gamma }{\lambda_{k}\left(1-\rho_{k}^{2}\right)}\right)-\\ & 2e^{-\frac{\gamma }{\lambda_{k}}}Q\left(\sqrt{\frac{2\gamma }{\lambda_{k}\left(1-\rho_{k}^{2}\right)}},\rho_{k}\sqrt{\frac{2\gamma }{\lambda_{k}\left(1-\rho_{k}^{2}\right)}}\right), \end{array}$$

where $\lambda_{k}=P\delta_{k}^{2}/N_{0}$, $Q\left(a,b\right)$ is the first order Marcum Q-fuction and $I_{0}\left(\cdot \right)$ is the modified Bessel function of the first kind of order zero [24]. Plugging Equation (16) into Equation (15) gives

(17) $$\begin{array}{cc} F_{\min}^{k}\left(\gamma \right) & =1-2e^{-\frac{\gamma }{\lambda_{k}}}-e^{-\frac{2\gamma }{\lambda_{k}\left(1-\rho_{k}^{2}\right)}}I_{0}\left(\frac{2\rho_{k}\gamma }{\lambda_{k}\left(1-\rho_{k}^{2}\right)}\right)\\ & +2e^{-\frac{\gamma }{\lambda_{k}}}Q\left(\sqrt{\frac{2\gamma }{\lambda_{k}\left(1-\rho_{k}^{2}\right)}},\rho_{k}\sqrt{\frac{2\gamma }{\lambda_{k}\left(1-\rho_{k}^{2}\right)}}\right). \end{array}$$

Additionally, the CDF of $\frac{1}{2}\min\left(\gamma_{SR_{k}},\gamma_{R_{k}D}\right)$, $F_{\frac{1}{2}\min}^{k}\left(\gamma \right)$, can be directly obtained as

(18) $$\begin{array}{c} F_{\frac{1}{2}\min}^{k}\left(\gamma \right)=F_{\min}^{k}\left(2\gamma \right). \end{array}$$

Now, let us define $\gamma_{L}$ and $\gamma_{U}$ as follows:

(19) $$\begin{array}{c} \gamma_{L}=\max_{k=1,\cdots ,K}\left[\frac{1}{2}\min\left(\gamma_{SR_{k}},\gamma_{R_{k}D}\right)\right], \end{array}$$

(20) $$\begin{array}{c} \gamma_{U}=\max_{k=1,\cdots ,K}\left[\min\left(\gamma_{SR_{k}},\gamma_{R_{k}D}\right)\right]. \end{array}$$

Then, it is readily to show that

(21) $$\begin{array}{c} \mathrm{Pr}\left[\gamma_{U}\leq \gamma \right]\leq \mathrm{Pr}\left[\gamma_{SR_{b}D}\leq \gamma \right]\leq \mathrm{Pr}\left[\gamma_{L}\leq \gamma \right]. \end{array}$$

By exploiting Equation (21), we can derive lower and upper bounds on the system outage probability as follows:

(22) $$\begin{array}{c} \prod_{k=1}^{K}F_{\min}^{k}\left(\gamma_{th}\right)\leq P_{out}\leq \prod_{k=1}^{K}F_{\min}^{k}\left(2\gamma_{th}\right), \end{array}$$

where $\gamma_{th}=2^{2R_{0}}-1$.

Although the lower and upper bounds on the system outage probability are useful for readily evaluating the system performance without doing complicated computer simulations, they are in general too complex to gain insight. Motivated by this, we now look into the high SNR region and derive asymptotic approximations for the lower and upper bounds, which enables the characterization of the achievable DO of the system. For that purpose, we will consider the case that ${\left\{\lambda_{k}\right\}}_{k=1}^{K}=\lambda \to \infty$ and ${\left\{\rho_{k}\right\}}_{k=1}^{K}=\rho$. Then, by using the Taylor approximation of the Marcum Q-function [25] (first order only), we obtain

(23) $$\begin{array}{c} Q\left(\sqrt{\frac{2\gamma_{th}}{\lambda \left(1-\rho^{2}\right)}},\rho \sqrt{\frac{2\gamma_{th}}{\lambda \left(1-\rho^{2}\right)}}\right)\approx 1-\frac{\rho^{2}\gamma_{th}}{\lambda \left(1-\rho^{2}\right)}, \end{array}$$

which holds if $\lambda \left(1-\rho^{2}\right)\gg 1$. Making use of Equation (23), we approximate $F_{\min}^{k}\left(\gamma \right)$ as follows:

(24) $$\begin{array}{c} F_{\min}^{k}\left(\gamma \right)\approx \frac{2\gamma_{th}}{\lambda }, \end{array}$$

where we have utilized $I_{0}\left(x\right)\overset{x\approx 0}{\to }1$ and the Taylor approximation of exponential functions. Using Equation (24), we can arrive at

(25) $$\begin{array}{c} P_{low}=\prod_{k=1}^{K}F_{\min}^{k}\left(\gamma_{th}\right)\approx {\left(\frac{2\gamma_{th}}{\lambda }\right)}^{K}, \end{array}$$

(26) $$\begin{array}{c} P_{up}=\prod_{k=1}^{K}F_{\min}^{k}\left(2\gamma_{th}\right)\approx {\left(\frac{4\gamma_{th}}{\lambda }\right)}^{K}, \end{array}$$

from which we can obtain

(27) $$\begin{array}{c} O\left(\lambda^{-K}\right)\approx P_{low}\leq P_{out}\leq P_{up}\approx O\left(\lambda^{-K}\right), \end{array}$$

which shows that the considered system can achieve a full DO equal to the number of relays.

For an extreme case when ρ=1, the source-relay $R_{k}$ and the relay $R_{k}$-destination channels experience the same fading. In other words, $\gamma_{SR_{k}}$ is equal to $\gamma_{R_{k}D}$. Consequently, the selected relay has not only the best source-relay channel but also the best relay-destination channel. The system in this case is similar to the Max-Max relay selection system [26], which is known to provide a full DO of *K* with an additional SNR gain compared to the conventional BRS systems.

## 5. Simulation Results

In this section, we provide numerical and simulation results to validate our analysis. The simulation setting follows the system model in Section 4 with $R_{0}=2$ bit/s/Hz, ${\left\{\lambda_{k}\right\}}_{k=1}^{K}=\lambda$, P=1, $N_{0}=1$ (hence, the received SNR will be lumped into the channel variances), and ${\left\{\rho_{k}\right\}}_{k=1}^{K}=\rho$.

**Figure 4:**
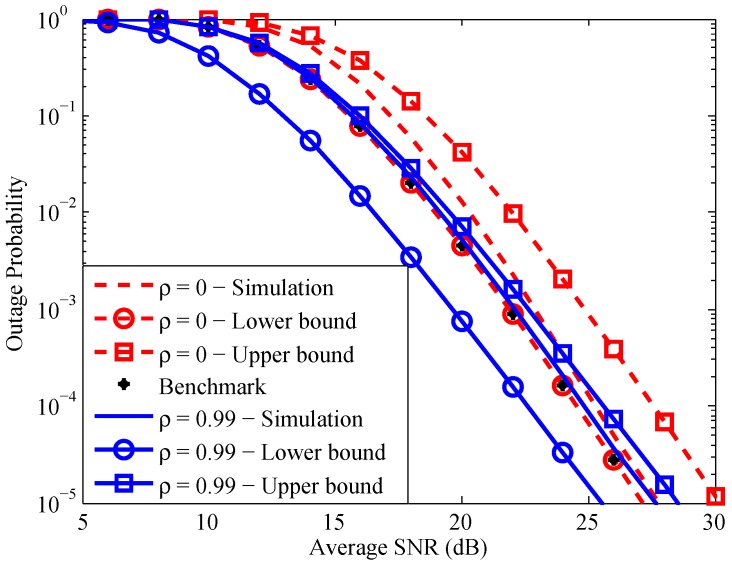
System outage probability versus average received SNR with K=4.

**Figure 5:**
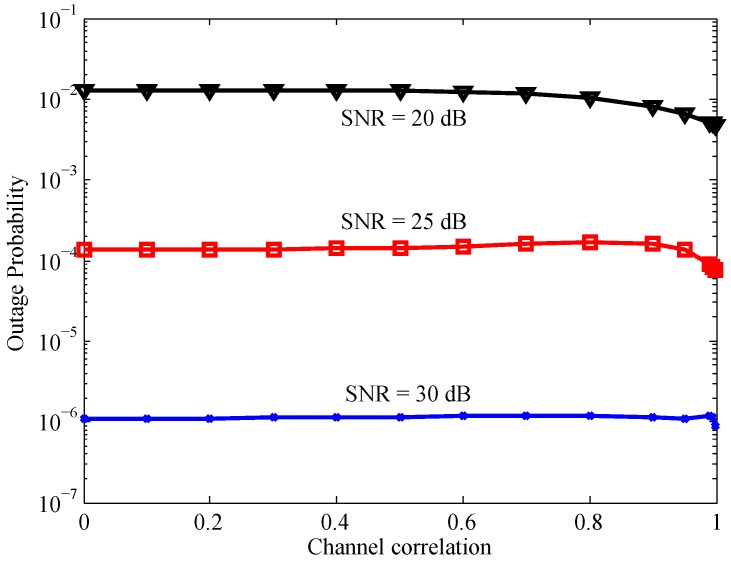
System outage probability versus average channel correlation with K=4.

Figure 4 illustrates the relation between the system outage probability and the average received SNR. As a benchmark comparison, we use Equation (6) of [27] with some appropriate modifications to obtain a lower bound on the outage probability of the system under independent fading channels. We first can see that when ρ=0, the derived lower bound is exactly the same with the benchmark lower bound for the independent channels case. It validates our derived bounds on the system outage probability. Secondly, when *ρ* is small (close to zero) the analytic lower bound is relatively tighter than the upper counterpart, while when *ρ* is close to one, the analytic upper bound is relatively tighter than the lower one. This observation infers that the correlation between two channels of each relay has a significant impact on the relation given in Equation (6). Moreover, it is worth noting that when the channel correlation coefficients increase, the system outage probability decreases in low and intermediate SNR range, while it stays the same in the high SNR region. This fact can be further validated through Figure 5, in which we simulate the system outage probability versus the channel correlation coefficient for several average SNR values.

Figure 5 shows that when the channel correlations approach one, the system outage probability decreases. One reasonable explanation may be described as follows. The end-to-end SNR at the destination via the relay $R_{k}$ is upper bounded by the minimum between the two single-hop SNRs, i.e., $\gamma_{SR_{k}}$ and $\gamma_{R_{k}D}$. Under the independent channel scenarios, when $\gamma_{SR_{k}}$ is large, $\gamma_{R_{k}D}$ can be small and mainly decides the system performance. In other words, when one of the single-hop SNRs is large, the system performance can be sometimes good and sometimes bad. However, when the channels are strongly correlated, if $\gamma_{SR_{k}}$ is large, $\gamma_{R_{k}D}$ will also be large with a high probability. As a result, the system performance will be good with a high probability. This observation implies that the considered correlation form has a constructive effect on the system performance. Note that a similar observation for a single DnF relay system under the same correlated environment has been recently presented in [15,28]. This is an important observation, which is in contrast to the existing results presented in [17,18,19,20,21], where channel correlations therein have a destructive effect on the system performance. In addition, it is noticed that an increase of the average SNR enlarges the threshold where the system outage probability starts decreasing. Particularly, in a high SNR range, for most values of *ρ*, the effect of channel correlation on the system performance is negligible.

**Figure 6:**
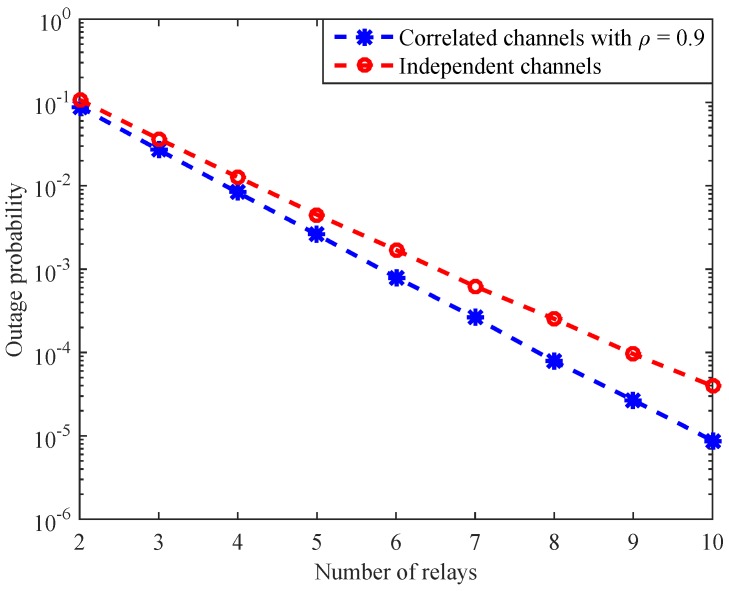
System outage probability versus the number of relays at 20 dB average SNR.

**Figure 7:**
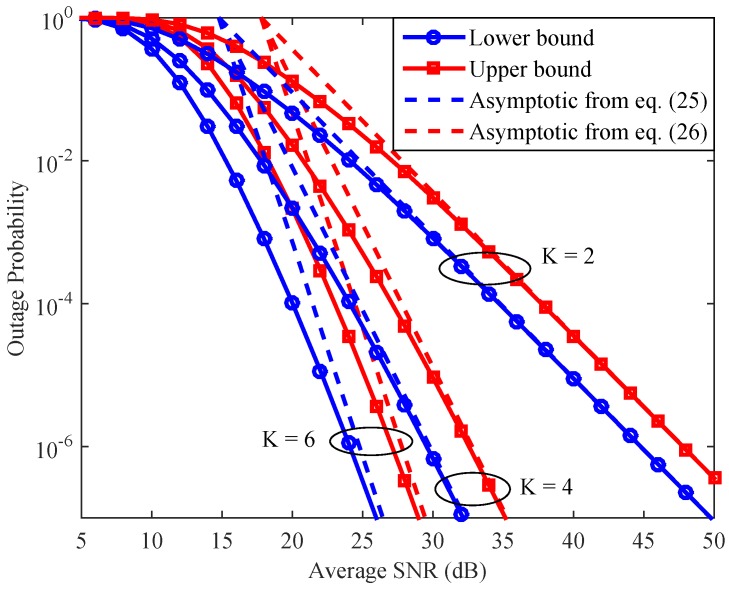
Upper and lower bounds on the system outage probability and their asymptotic approximations with ρ=0.9.

To illustrate the effect of multiple relays on the system performance, we present Figure 6 and Figure 7. In Figure 6, we simulate the system outage probability versus the number of relays at 20 dB average SNR for independent channels and correlated channels with ρ=0.9. The figure first shows that increasing the number of relays significantly improves the system performance, as expected. Moreover and importantly, it is observed that when the number of relays increases, the gap between the system performance under correlated channels and that under independent channels is enlarged. The reason is that the greater the number of relays, the greater the correlation coefficients we have. Since the correlations are beneficial for the system, the system performance is enhanced.

Figure 7 shows the upper and the lower bounds on the system outage probability and their asymptotic approximations given in Equations (25) and (26) for several values of *K*. It is noteworthy that the curves generated by Equations (25) and (26) represent the system DO. We can see that the DO curves converge to their corresponding lower and upper bound curves in the high SNR region, which confirms that the considered system can achieve DO equal to the number of available relays. In addition, the figure indicates that, by implementing a greater number of relays, the system can obtain a much better outage performance. The reason is that with a greater number of relays, the system has more freedom to select the best helper for its communication.

**Figure 8:**
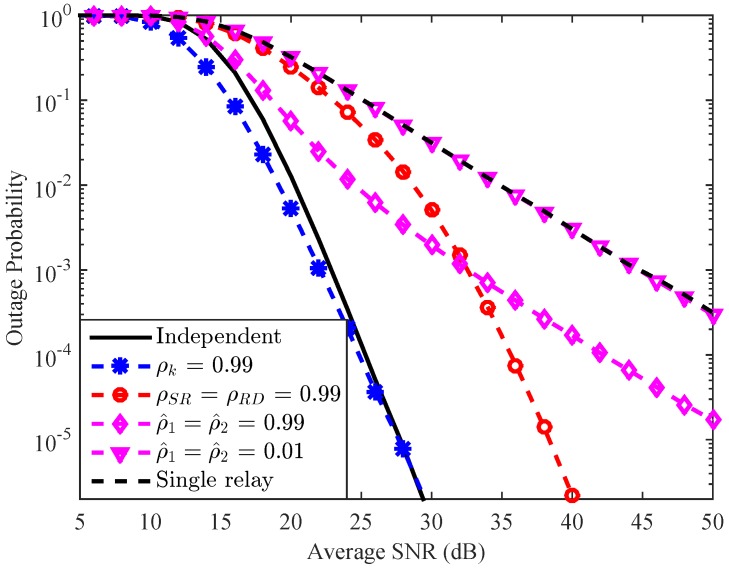
A comparison among the effects of several channel correlation forms on the system performance with K=4.

In Figure 8, we present a comparison among the effects of the correlations between a channel and its estimate $\left\{\rho_{1},\rho_{2}\right\}$, correlations among the first-hop channels and correlations among the second-hop channels $\left\{\rho_{SR},\rho_{RD}\right\}$, and the correlation between two channels of each relay $\left\{\rho_{k}\right\}$ on the considered system performance. The ’independent curve’ also represents the case with perfect channel estimation. It is again confirmed that among the three correlation forms, only the correlation between the two channels of each relay has a positive impact on the system performance, even though the improvement of the system outage probability is diminished in the high SNR region. We observe that the dash curve with circle mark and the dash curve with asterisk mark are parallel with the solid curve in the high SNR region. It means that the last two aforementioned correlation forms do not affect the system achievable DO [21]. On the other hand, it is shown that the two dash curves with diamond and triangle marks are parallel with the dash curve in the high SNR region. This observation infers that the first aforementioned correlation form significantly degrades the system DO, i.e., from four to one [18,19]. It is also worth mentioning that even with ${\hat{\rho }}_{1}={\hat{\rho }}_{2}=0.99$ (this corresponds to the case that the estimate channels are only very little outdated compared to the actual channels), the system performance as well as the system DO are seriously deteriorated. This observation implies that the full benefit of single relay selection scheme is difficult to achieve in practise since perfect channel estimation is not an easy task to be done.

## 6. Conclusions

In this work, we studied the impact of the channel correlations on the performance of AnF cooperative systems. We first briefly reviewed existing works on channel correlations. We then focused on analyzing the effect of the correlation between two channels of each relay on the performance of a multiple AnF relays system with the BRS scheme. Based on the lower and upper bound of the end-to-end received SNR at the destination, we derived the corresponding upper and lower bounds on the system outage probability. In addition, and importantly, we proved that the system can achieve a full DO equal to the number of relays. Moreover, we showed an interesting result that can be stated as “when the considered channel correlation coefficient increases, the system outage probability decreases”.

The scope of future research in this subject is quite broad. Hence, we now would like to discuss only a few interesting and challenging problems which are worth further consideration. Up to now, the equally correlated model, in which correlation coefficients are the same, has been largely investigated [17,18,19,20,21]. Even though the equally correlated model could be considered as a worst/best-case benchmark and its related results help us to gain fundamental knowledge of the effect of the channel correlations on the performance of cooperative systems, it may fail to precisely predict the actual performance of cooperative systems in reality. To understand the true impact of the channel correlations, non-identical correlated models should be taken into account. Such a study in combination with existing results will create a more complete description of the effect of the channel correlations on the performance of relaying systems.

On the other hand, most existing works are focused on studying the performance/behaviour of cooperative systems under a single channel correlation form only, but have ignored the joint consideration of different forms of channel correlation. Although [15,29] investigate the joint effect of the correlations between source-destination and source-relay channels, between source-destination and relay-destination channels, and between source-relay and relay-destination channels on the performance of DnF systems, only a single relay configuration is considered. In addition, the presented symbol error probability given in [15,29] cannot be straightforwardly extended to multi-AnF-relay scenarios. Moreover, jointly consider inter-path correlation and correlation between a channel and its outdated estimate is also an interesting problem. Therefore, theoretical analyses and simulations are required to reveal novel insights on the joint effect of multiple channel correlation forms considering multiple AnF relays.

With recent advances in antenna design and signal processing, the full-duplex (FD) relaying mode has gained more and more attractiveness from the research community. The advantages of the FD relaying mode lies in the fact that it can address the inefficient use of resources, i.e., dedicated bandwidth or time-slot, of the half-duplex counterpart. Due to its advantages, applications based on FD relay systems will be widely spread in the near future. Hence, investigating the impact of the channel correlations on the performance of FD cooperative systems is necessary and important. In addition, taking the self-interference link between a relay’s input and output in FD cooperative systems into account will make the related problems much more challenging.

Finally, we note that although the broadcast nature of wireless medium greatly facilitates cooperative communication, it makes wireless data transmission vulnerable to eavesdropping attack. In addition, beside complex cryptography at the network layer, physical layer security (PLS) is emerging as a new paradigm to robust cooperative systems security level against eavesdroppers by exploiting characteristics of wireless channels and noise [30,31]. The advantage of PLS is that it can operate independent of higher layers, so that it can be used to augment existing security schemes. PLS quantifies a system secrecy by secrecy capacity, which basically is the maximum between ’zero’ and ’the difference between capacity obtained at a legitimate destination and capacity obtained at eavesdroppers’. Since the secrecy capacity is usually in complex form, investigating the effect of the channel correlations on the secrecy performance of cooperative systems is a research-worthy, yet challenging, problem.

## Figures and Tables

**Table 1:** A summary of channel correlation forms.

Channel Correlation	Description
${\hat{\rho }}_{ij}$	Correlation between the actual channel ($h_{ij}$) and the corresponding estimate ${\hat{h}}_{ij}$
$\rho_{k}$	Correlation between two channels of the relay $R_{k}$
$\rho_{SD}^{SR_{k}}$	Correlation between $h_{SR_{k}}$ and $h_{SD}$ channels
$\rho_{SD}^{R_{k}D}$	Correlation between $h_{R_{k}D}$ and $h_{SD}$ channels
$\rho_{SR_{k}}^{SR_{l}}$	Correlation between the first-hop channels
$\rho_{R_{k}D}^{R_{l}D}$	Correlation between the second-hop channels

**Table 2:** A summary of channel correlation forms which have been investigated.

Model	Reference	${\left\{{\hat{\rho }}_{SR_{k}}\right\}}_{k=1}^{K}$	${\left\{{\hat{\rho }}_{R_{k}D}\right\}}_{k=1}^{K}$	${\left\{\rho_{SR_{k}}^{SR_{l}}\right\}}_{k=1}^{K}$	${\left\{\rho_{R_{k}D}^{R_{l}D}\right\}}_{k=1}^{K}$	${\left\{\rho_{k}\right\}}_{k=1}^{K}$	${\left\{\rho_{SD}^{SR_{k}}\right\}}_{k=1}^{K}$	${\left\{\rho_{SD}^{R_{k}D}\right\}}_{k=1}^{K}$
A	[17,18,19]	${\hat{\rho }}_{1}$	${\hat{\rho }}_{2}$	0	0	0	0	0
B	[20]	1	1	0	0	$\rho_{R}$	$\rho_{SD}^{SR}$	$\rho_{SD}^{RD}$
C	[21]	1	1	$\rho_{SR}$	$\rho_{RD}$	0	0	0
